# The Localized Neuroendocrine Transformation of Prostate Adenocarcinoma: A Case Report and Literature Review of Current Treatment Modalities

**DOI:** 10.7759/cureus.60790

**Published:** 2024-05-21

**Authors:** Anna Eckrich, Ana Colon, Lilia Davenport, Shreya Goyal

**Affiliations:** 1 Pharmacy, The Brooklyn Hospital Center, New York City, USA; 2 Oncology, The Brooklyn Hospital Center, New York City, USA

**Keywords:** small cell carcinoma, neuroendocrine prostate cancer, neuroendocrine transformation, prostate cancer, small cell carcinoma of the prostate

## Abstract

Most prostate cancers are adenocarcinomas. However, there is a rare and aggressive subtype known as small cell carcinoma of the prostate (SCCP). This variant of prostate cancer is marked by its distinctive features, including high-grade malignancy, neuroendocrine differentiation, and a unique clinical presentation, often involving metastases. This report details the presentation and management of a 66-year-old African-American male who was originally diagnosed with high-risk adenocarcinoma of the prostate. At initial diagnosis, the patient was suboptimally treated with radiation alone without androgen deprivation therapy (ADT). On re-biopsy several years later, he was found to have localized recurrent disease with transformation into SCCP. The prognosis for SCCP is poor with a mean survival. Patients typically present with metastases, commonly to the brain, liver, bones, or bladder. SCCP after treatment for adenocarcinoma of the prostate is more common than de novo presentation. The amount of neuroendocrine differentiation of SCCP often increases with treatment, particularly after treatment with ADT. This report emphasizes the importance of timely and optimal care when treating prostate cancer and suggests potential consequences that inappropriate treatment or treatment delays may entail.

## Introduction

Prostate cancer is a commonly diagnosed malignancy among men worldwide. Most prostate cancers are adenocarcinomas. However, there exists a rare and highly aggressive subtype known as small cell carcinoma of the prostate (SCCP). This subtype is considered to be a rare and aggressive form of cancer and accounts for less than 2% of all prostate cancers [1]. This variant of prostate cancer is marked by its distinctive features, including high-grade malignancy, neuroendocrine differentiation, and a unique clinical presentation [1]. Despite the severity of this disease, studies in the treatment for this specific disease are lacking and are extrapolated from the data available for the treatment of small cell carcinoma of the lung. This case report details the presentation and management of a patient who developed SCCP transformation after suboptimal treatment of a prior prostatic malignancy with adenocarcinoma histology and a review of previous literature published in SCCP. 

## Case presentation

A 66-year-old African-American male, with a past medical history of hypertension and dyslipidemia, was initially diagnosed with prostate cancer in early 2014 following hospitalization. His prostate-specific antigen (PSA) was 25 ng/mL, and biopsy and imaging were consistent with grade group 5, with no evidence of metastases (T1c cN0, cM0), placing the patient in the very high-risk category of localized prostate cancer [2]. Treatment was recommended with either surgery or radiation along with androgen deprivation therapy (ADT). He declined surgery and ADT and agreed to receive external beam radiation therapy, despite understanding that this approach would be suboptimal to achieve a cure and a durable remission, which was completed at the end of January 2015. The PSA downtrended to a nadir of 1.5 ng/mL in late 2015. 

**Figure 1:**
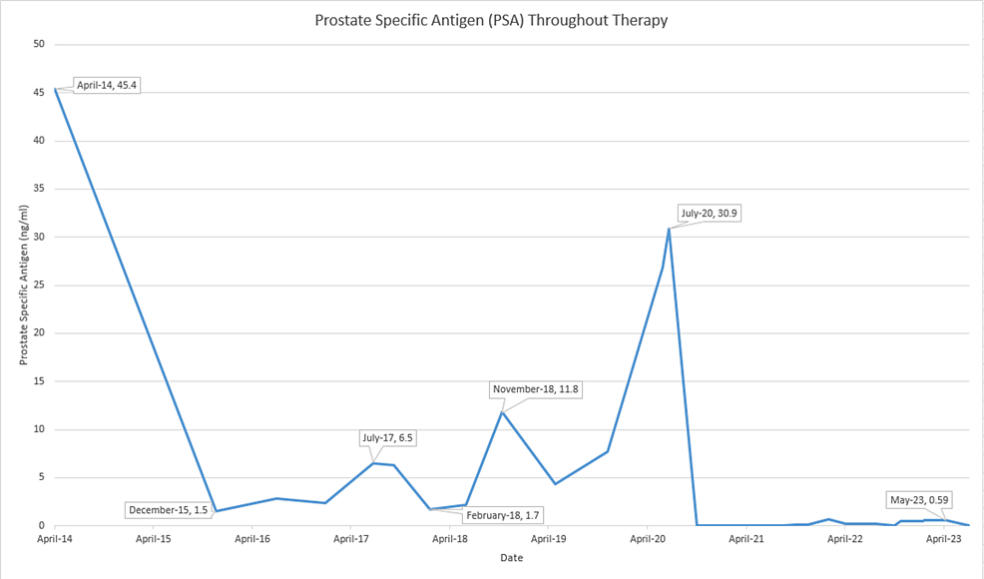
PSA (ng/mL) throughout therapy PSA: prostate-specific antigen

In July 2017, PSA was noted to have risen significantly to 6.5 ng/mL. Adherence issues were of major concern with this patient; he frequently missed appointments. In six months, PSA continued to trend up to a level of 11.8 ng/mL (Figure 1).

In June 2020, PSA had risen to 26.8 ng/mL. Restaging imaging did not show any evidence of metastases and was deemed to be suboptimally treated for his very high-risk prostate cancer as opposed to having biochemical recurrence. 

**Figure 2:**
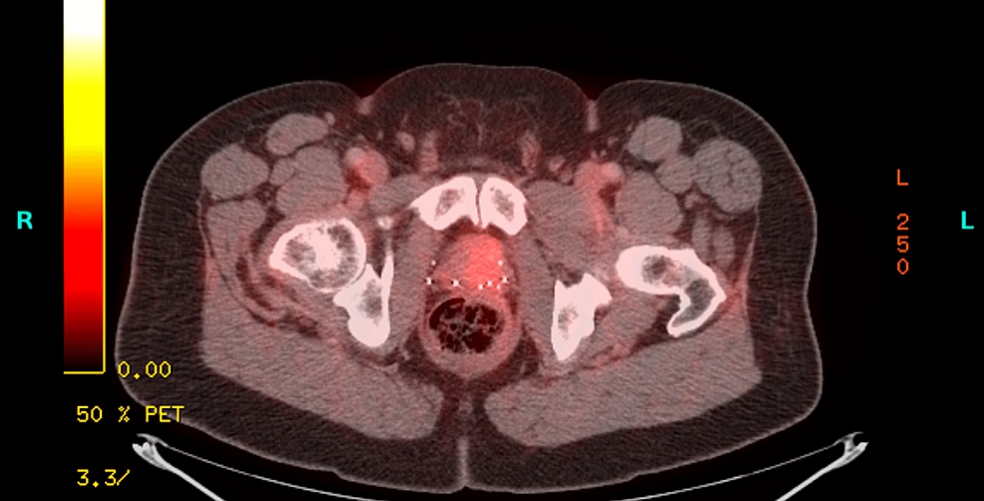
PET-CT, a selected axial cross-section at the level of the prostate from July 8, 2022

Treatment with ADT was initiated with the plan of continuing for three years. While on ADT, his PSA remained at less than 0.01 ng/mL from October 2020 to September 2021. In December 2021, there was a rise in the PSA to 0.1 ng/mL, which continued having subtle increases in subsequent visits but remained below biochemical recurrence levels. At the end of 2022, his PSA level rose to 0.2 ng/mL, and a pelvic MRI was performed, which revealed a 2.2 cm lesion with a very high likelihood of recurrence. PET-CT did not show any signs of metastatic disease (Figure 2). 

**Figure 3:**
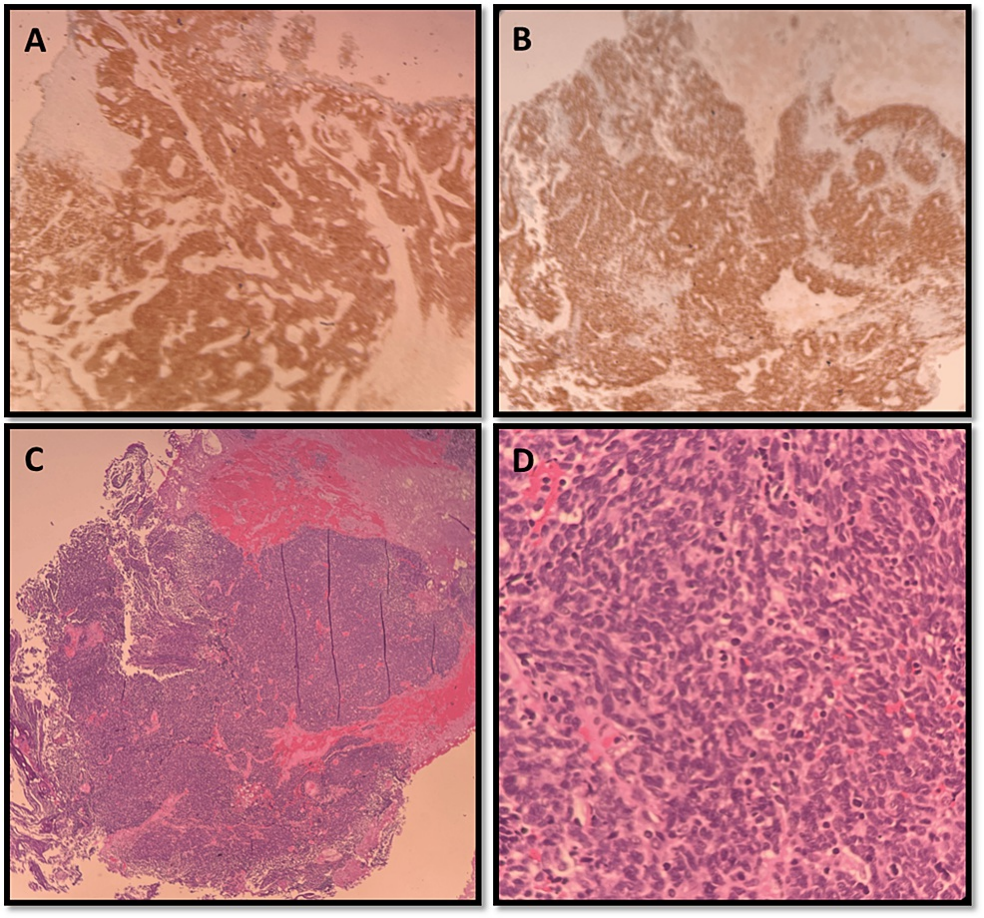
Prostate biopsy A: positive synaptophysin corresponds to prostatic tissue histology; B: positive Ki-67; C: H&E (hematoxylin and eosin) staining showing clusters of small blue cells; D: high power field, similar to image C, showing small round blue cells characteristic in poorly differentiated neuroendocrine carcinoma

Multiple follow-up appointments were missed before the patient reported worsening urinary frequency and urgency prompting a cystoscopy, which showed evidence of clots at the bladder neck. Transurethral resection of bladder tumor (TURPT) and transurethral resection of the prostate (TURP) were scheduled at that time, but the patient delayed both procedures until June 2023. When finally performed, the pathology of the biopsied prostatic lesions showed small cell carcinoma. Biomarker testing of the new biopsy was positive for androgen receptor (AR) mutation, retinoblastoma susceptibility gene (RB1) mutation, and moderate proliferative activity of Ki67 at 70-80%. A second opinion from a tertiary care center confirmed the diagnosis of small cell/high-grade neuroendocrine carcinoma, with focal areas of prostatic acinar adenocarcinoma, grade group 5 (Figures 3A-3D). With a change in diagnosis, restaging with PET-CT and MRI brain was done again, and both tests showed negative results for distant metastases.

Following guidelines for small cell cancer of the lungs, the patient planned to be started on cisplatin and etoposide. Four cycles were planned, with cisplatin 75 mg/m^2^ on day 1 and etoposide 80 mg/m^2^ on days 1-3, repeated every 21 days. The patient refused port placement but received his first cycle of treatment successfully on August 29, 2023. The second cycle was attempted on September 26, 2023; however, this cycle was aborted on day 2 due to the inability to obtain IV access and concerns regarding spinal cord involvement, with increased episodes of urinary incontinence. Further treatment was delayed due to the patient being adamant about pausing treatment for a several-month-long trip overseas. 

Following his return in January 2024, he came to the clinic with no acute complaints. He continued to refuse IV access or placement of a port, as well as the continuation of chemotherapy, despite knowing the risk of progression and metastases or death. At this visit, PSA was noted to have risen to 0.54 ng/mL from 0.13 ng/mL in August 2023. The patient was amenable to PET-CT, which continues to show no signs of metastases to this date.

## Discussion

In the United States alone, there are estimated to be over 288,000 new diagnoses of all subtypes of prostate cancer with almost 35,000 deaths [3]. The prognosis for SCCP is grim compared to its adenocarcinoma counterpart. After diagnosis, patients have a mean age of 70 years old and a mean survival of only five months [4]. SCCP is an aggressive disease, and more than half of all patients will typically present with metastases upon diagnosis commonly to the brain, liver, bones, and bladder [4,5]. Unfortunately, the true incidence of SCCP is likely underestimated. Autopsy reports have shown that 10-20% of patients with castration-resistant prostate cancer had developed SCCP, with an ever higher incidence rate in those with metastatic disease [6]. Additionally, prostate cancer may not be on the differential as patients present with a normal PSA, further explaining low incidence levels [4]. Unlike adenocarcinoma of the prostate, patients with SCCP may present with low to normal PSA levels, even with a large tumor burden [7].

The SCCP was first reported almost 50 years ago; however, little advancement has been made in its management [4]. Most treatment approaches draw parallels with the treatment of small cell lung cancer due to the shared neuroendocrine characteristics between the two types [8]. First-line regimens involve cytotoxic chemotherapy with a platinum agent, including combinations such as cisplatin and etoposide, carboplatin and etoposide, carboplatin and docetaxel, and carboplatin and cabazitaxel [2]. As prostate cancer that has progressed to small cell carcinoma lacks AR, there is typically no place in therapy for ADT unless patients are presenting de novo, and the biopsy has a large acinar adenocarcinoma component [1].

SCCP after treatment for adenocarcinoma of the prostate is more common than a de novo presentation [9]. Other notable mutations common in SCCP include RB1 mutations as well as amplification of other neuroendocrine markers such as Aurora kinase A (AURKA) and neuroblastoma-derived myc oncogene (N-myc) [6,9]. AURKA amplifications have been identified in approximately 65% of prostate adenocarcinoma that evolve into neuroendocrine tumors, including small cell, but only present in 5% of pure adenocarcinoma tumors, suggesting a potential marker or target for small cell prostate cancer or used to predict disease aggressiveness [6,10].

Immunotherapy has been used in SCCP with varying results. Immunotherapy, with atezolizumab and durvalumab, has shown efficacy when used in combination with chemotherapy in small cell lung cancer [8]. However, a study found no benefit in adding immunotherapy in this population. Wee et al. evaluated the use of atezolizumab in addition to carboplatin and etoposide. They found a median overall survival of 3.4 months, indicating no difference from the current standard of care [11]. However, the limitations of this study include a small sample size (n = 7) and a retrospective nature [11]. The limited efficacy of immunotherapy in neuroendocrine prostate cancer was also shown in a case series analyzed by Pokhrel et al. Two cases were presented: one with adenocarcinoma with foci of neuroendocrine prostate cancer, and the other had adenocarcinoma that transformed after treatment with ADT [12]. Both cases received immunotherapy with durvalumab and showed only limited efficacy [12]. 

A novel class of anticancer drugs called AURKA inhibitors may have promising results in SCCP. AURKA amplification has been shown to drive neuroendocrine differentiation, and inhibiting this pathway may be beneficial [13]. AURKA inhibitors, such as alisertib, inhibit the interaction between N-myc and AURKA to stop tumor growth and progression [14]. A phase II trial evaluated 60 patients with metastatic prostate cancer who were then treated with alisertib [14]. This study did not meet its primary endpoint of progression-free survival; however, it identified patients who responded significantly to AURKA inhibitors [14]. Some classes of responders identified include those with extensive visceral metastasis and those with platinum-refractory disease, indicating the need for future studies in these populations [14].

The amount of neuroendocrine differentiation of SCCP often increases with treatment, particularly after treatment with ADT [9,15]. The incidence of treatment-related neuroendocrine small cell prostate cancer is increasing due to the development of more potent AR inhibitors used in ADT as a method to avoid AR pathway inhibition [13]. Once progressed, tumor cells no longer express AR as a mechanism of resistance to previous ADT [6,9]. Progression has also been described after the treatment of adenocarcinoma of the prostate with brachytherapy [15]. However, the neuroendocrine differentiation after suboptimal treatment of adenocarcinoma of the prostate in high-risk patients has not been reported and requires attention. More studies are needed to determine whether non-compliant patients have an increased risk of transformation into a more aggressive form of prostate cancer such as SCCP. 

## Conclusions

This case presents the management of a 66-year-old man initially diagnosed with very-high-risk adenocarcinoma of the prostate that then transformed into SCCP. This patient’s treatment course illustrates the significance of how suboptimal treatment can have profound consequences later in a treatment course. Failure to initiate ADT and failure of the patient to be compliant with treatment consistently and maintain scheduled appointments are factors that could have allowed his cancer to progress to SCCP with neuroendocrine transformation. This case also shows a potential association between initial risk stratification and the risk of transformation into neuroendocrine cells. SCCP is an aggressive and rare subtype of prostate cancer. After transformation, tumor cells become resistant to conventional treatments for adenocarcinoma such as ADT. This subtype of prostate cancer has been theorized to be associated with adenocarcinoma that has been treated previously with ADT. With this transformation, typically comes rapid progression as represented by the presence of metastases, particularly to the brain, liver, bones, or bladder. However, in this particular case, the patient did not develop metastases at the initial diagnosis of SCCP. The potential for adenocarcinoma of the prostate to transform into SCCP emphasizes the need for further education for both providers and patients on appropriate initial treatment as well as proper monitoring and follow-up.

SCCP presents a challenge due to limited treatment options. Research involving new advancements in small cell lung cancer as well as with the development of a new class of agents have been inconclusive with SCCP. While current therapeutic strategies are similar to small-cell lung cancer treatments, research for effective and prostate-cancer-specific treatment continues. The association of suboptimal or delayed care and transformation to SCCP is currently unclear but prompts future studies and increased awareness for healthcare providers to ensure they are delivering high-quality care and educating patients on compliance and medication adherence to ensure proper treatment and to avoid undesired outcomes. This case reinforces the need for improved patient and healthcare provider education, consistent follow-up, and further research in the progression and treatment of SCCP.
